# Elective admissions and treatments in the final three months of life: a nationwide retrospective study of older adults

**DOI:** 10.1007/s41999-026-01560-5

**Published:** 2026-08-03

**Authors:** Sofie Rutten, Joost W van den Berg, Rob D van der Mei, Bianca M Buurman, Hanna C Willems

**Affiliations:** 1 Section of Geriatric Medicine, Internal Medicine, Amsterdam University Medical Centre Location University of Amsterdam, Meibergdreef 9, 1105 AZ Amsterdam, The Netherlands; 2 Professional Performance and Compassionate Care, Department of Medical Psychology, Amsterdam University Medical Centre Location University of Amsterdam, Amsterdam, The Netherlands; 3 Stochastics, Centrum Wiskunde & Informatica, Amsterdam, The Netherlands; 4 Medicine for Older People, Amsterdam University Medical Centre Location Vrije Universiteit, Amsterdam, The Netherlands; 5 Amsterdam Movement Sciences Institute, Amsterdam University Medical Centre Location Vrije Universiteit Amsterdam, Amsterdam, The Netherlands

**Keywords:** Aged, Aged, 80 and over, Palliative care, Retrospective studies, Registries, Elective surgical procedures

## Abstract

**Purpose:**

Research on potentially inappropriate care at the end of life in older adults rarely distinguishes between acute and elective care. As elective care is plannable, it allows for timely deliberation on its appropriateness. This study aimed to explore elective hospital care and specific elective treatments in the final three months of life to identify areas warranting re-evaluation.

**Methods:**

A nationwide retrospective observational study was conducted using linked administrative data, including data from electronic patient records and health insurance claims from all secondary and tertiary hospitals across the Netherlands. Included were 104,544 older adults aged ≥ 65 who died between April and December 2019.

**Results:**

In the final three months of life, 41.7% of older adults received no hospital care, 14.5% received outpatient care only, 42.4% were admitted, and 15.9% were electively admitted. Some of the most frequent elective treatments were chemo- and/or immunotherapy (12,102), radiotherapy (13,924), dialysis (30,087), and cataract surgery (584). Treatments during elective admissions included gastrointestinal endoscopies (979), gastrointestinal tract organ resections (229), gastrointestinal-ostomy surgeries (228), exploratory laparotomies (177), heart valve surgeries (179), arterial interventions (coronary: 266; aorta/side branches: 147; peripheral: 786), hip surgeries (136), leg amputations (115), and artificial feeding procedures (683). A substantial proportion occurred in the final month of life.

**Conclusion:**

Elective hospital care is common in the final three months of life among older adults, with limited time to experience meaningful benefit. These findings highlight the need to reconsider the appropriateness of elective care at the end of life to better align care with patients’ preferences.

**Supplementary Information:**

The online version contains supplementary material available at https://doi.org/10.1007/s41999-026-01560-5.

## Key summary points

**Aim:**

To explore elective hospital care and specific elective treatments in the final three months of life of older adults and identify areas warranting re-evaluation.

**Findings:**

16% of older adults were electively admitted in the final three months of their lives. During these admissions invasive and intensive procedures took place.

**Message:**

Given that elective hospital care is not uncommon in the final months of life, its plannable nature should be utilized to integrate prognostic assessment and shared decision‑making, promoting care that aligns with patients’ end‑of‑life goals and reduce inappropriate interventions.

**Supplementary Information:**

The online version contains supplementary material available at https://doi.org/10.1007/s41999-026-01560-5.

## Introduction

In the final months of life, many older adults receive potentially inappropriate care [1–7]. This highlights the urgent need to re-examine current health care practices for this population, particularly in light of an ageing population, rising rates of multimorbidity, and an increasing need for palliative care [8–10].

Inappropriate end-of-life care refers to medical treatments and interventions provided near the end of life where benefits no longer outweigh possible negative effects of the treatment. This often involves prioritizing life-prolonging measures over comfort-oriented care, even when such interventions are not consistent with the patient’s prognosis or preferences. Instead, end-of-life care should aim to maximize the patient's remaining quality of life and relieve physical, psychosocial, and spiritual suffering, ensuring that support and medical decisions respect individual autonomy and align with personal goals [11, 12].

To date, frameworks for investigating inappropriate care in the end-of-life phase have predominantly focused on broad indicators, for example, hospitalizations, intensive care admissions, systemic therapies, and life-sustaining interventions [1–6]. To our knowledge, research has not specifically addressed elective care during this phase, nor has inappropriate end-of-life care been explicitly differentiated according to treatment urgency. This underrepresentation is important, as elective treatments equally have the potential to negatively influence care trajectories, financial costs, outcomes, and quality of life. Elective treatments may do more harm than good when the remaining lifespan is limited, with risks and complications possibly outweighing benefits, complicated rehabilitation detracting from quality of life, and resources being misallocated. Inappropriate elective care may even accelerate the process of dying. Yet, elective treatments are, by definition, plannable. As such, they allow for careful assessment of prognosis, timely weighing of advantages and disadvantages, informed discussions between patient and physician, and ultimately, proper shared decision making. Thus, gaining knowledge specifically about elective treatments executed at the end of life can initiate re-evaluation of these treatments and reduce them when inappropriate.

This study aimed to gain insight into potentially inappropriate elective treatments in the final three months of life of older adults. To achieve this objective, we conducted a retrospective nationwide study utilizing administrative data from Statistics Netherlands [25].

## Methods

### Study design an data sources

For this explorative retrospective population-based observational study, we used routinely collected administrative data concerning persons who were aged ≥ 65 and died in 2019. Population-based retrospective cohort designs using administrative health data are commonly employed to examine healthcare utilization near the end of life [3, 6, 13–15]. We chose 2019 because post-COVID-19 pandemic data were incomplete.

Administrative registries accessed contained data on sociodemographic information, death certificates, received home care, hospital admissions, and claimed in- and outpatient hospital care (Online Resource 1.1). Admission data from Dutch Hospital Data covers all general and academic hospitals, and includes admission dates, wrong-bed days, admission type (one-day, clinical, observational), and emergency department visit on admission day. A ‘wrong-bed’ is a situation in which a person is clinically out of treatment but temporarily has no place to transfer to, for example, a nursing home. Claimed in- and outpatient hospital care data consist of claimed care activities and their dates. Care activities are provided by health care professionals in response to a patient’s healthcare demand and are fitted into Diagnosis Treatment Combinations (DTC). Dutch health care costs are paid in DTCs of which prices are fixed, based on the estimation of the average costs of an event.

The data were linked in Statistics Netherlands’ secure environment using a person-specific, dimensionless identification number. Reporting of this study followed STROBE and RECORD guidelines [16, 17].

### Study population

Persons aged ≥ 65 on 1 January 2019 who died between 1 April and 31 December 2019 were included. This window was chosen to ensure availability of data on hospital care in the final three months of life as data is supplied per calendar year. Patient characteristics from the geriatric domains, functional ability, physical health, cognition, psychological health, and socioenvironmental situation, were included to characterize our study population when possible (Online Resource 1.2, Table S1). The data on sex, migration background, living alone, income, medication dispensaries, home care, institutionalization, and causes of death were retrieved from Statistics Netherlands [25]. Data on income and medication dispensaries were unavailable for individuals institutionalized under the Long-Term Care Act. Income was reported at household level, which is not applicable to institutionalized persons, and medication records did not include drugs dispensed during hospital stays or provided by nursing homes.

### Outcomes

The primary outcomes were the 15 elective treatments occurring in the final three months of life and their frequency, proportion occurring in the final month of life, and number of individuals undergoing treatments. The final three months of life were defined as the end-of-life period, as the expected time to benefit from invasive treatments often exceeds this timeframe, consistent with previous population-based studies examining hospital care near death. The selection process is described in detail in subsequent sections and was based on the identification of in- and outpatient hospital care utilization, with a specific focus on elective admissions during the final three months of life. For our primary outcome, we focused on treatments during identified elective admissions as elective treatments with significant clinical impact are more likely to occur during admissions.

#### In- and outpatient hospital care utilization

Persons were classified as receiving any in- and outpatient hospital care when at least one care activity was declared within the final three months of life (Online Resource 1.3, Figure S1). This group was divided into persons receiving outpatient care only and persons who were admitted to the hospital. Hospital admissions were included when the discharge date was on or after the start of the final three months of life. ‘Wrong bed’ days were deducted from the end date, creating the discharge date, or start date if they occurred at the start of admission. Persons who were admitted were categorized into two groups: single admission or multiple admissions. As administrative data do not capture clinical intent of individual treatments, we defined admissions as elective when there was no corresponding emergency department visit or urgent face-to-face contact in another hospital department on the day of or prior to admission. This approach assumes that acute admissions are likely to involve an emergency department visit, whereas elective admissions typically do not.

#### Elective treatments

**Fig. 1:**
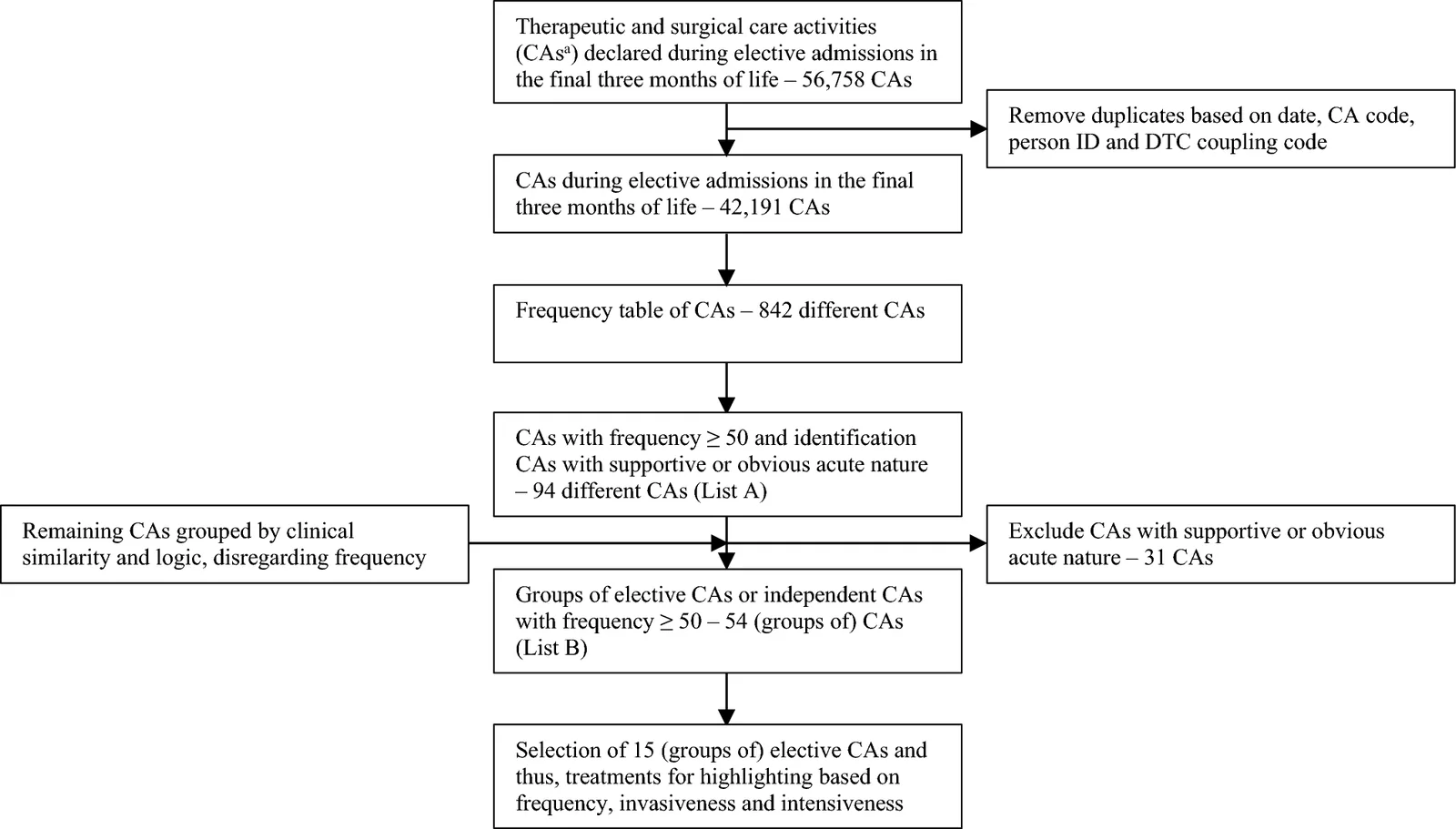
Flowchart of selecting frequent, intensive and/or invasive elective treatments to highlight. Online Resource 2, Table S2 and S3 contain full lists of care activities in list A and B. ^a^CAs: care activities. CAs during elective admissions in the final three months of life were selected, duplicates were removed, and a frequency table was generated. Frequent (≥50) CAs were extracted and treatment groups were created based on clinical similarity. 15 elective treatments were chosen to highlight based on frequency, invasiveness and/or intensiveness.

Unlike prior studies that operationalized potentially inappropriate end-of-life care using predefined quality indicators measured in the last month(s) of life, our objective was to explore the occurrence and nature of elective hospital care and invasive elective treatments; therefore, no appropriateness labels or established indicator sets were applied. Figure 1 outlines the steps taken to select 15 elective treatments. Duplicates of care activities during elective admissions declared on the same day, for the same person, within the same DTC sub-trajectory were removed. SR generated a frequency list of care activities with a therapeutic or surgical nature recorded during elective hospital admissions. Care activities with a frequency of ≥50 were extracted as a starting point, as infrequent treatments were unlikely to represent systematic elective practices. SR supplemented the list, in concordance with JB and HW, with additional care activities grouped based on clinical similarity regardless of individual frequencies. We determined the proportion of treatments occurring in the final month of life and the number of individuals per treatment, and reported the use of open versus minimally invasive approaches. SR, JB and HW then selected 15 treatments that were considered invasive, intensive, or relatively frequently performed, and likely to represent elective interventions. The selection reflects a balance between capturing the diversity of elective treatments and focusing on those most likely to be clinically significant. A more restrictive list would have necessitated more subjective decisions about which treatments to exclude; a more exhaustive list would have made for an incomprehensibly large paper.

### Statistical analysis

Data management and analyses were executed in R studio (version 2024.12.01). Baseline characteristics were summarized as medians (IQR) for non-normal distributions and counts (percentages) for categorical variables. Institutionalization in medication-related variables and missing values were treated as separate categories. The population was stratified by being electively admitted at least once (package: tableone). Groups were compared using Mann–Whitney *U* tests for continuous variables and Chi-square tests for categorical variables. A Sankey plot (NetworkD3; SankeyMATIC) visualized in- and outpatient hospital care in the final three months of life. Care activity frequencies were generated in R studio and grouped in Microsoft Excel (Microsoft Corporation) for treatment selection.

### Ethics approval and consent to participate

This study used pseudonymised administrative data from Statistics Netherlands. According to Dutch law and Statistics Netherlands regulations, the use of these data for scientific research does not require separate ethics approval, nor informed consent from individuals. The data were accessed within a secure environment and Statistics Netherlands provided guidelines for use and output control.

### Data sharing

Results of this study are based on calculations by Amsterdam University Medical Centre in project number 3056 using non-public microdata from Statistics Netherlands. All output of the current study is included in this article. Under certain conditions, these microdata are accessible for statistical and scientific research. For further information search: Microdata: Conducting your own research | CBS.

## Results

### Study population

**Fig. 2:**
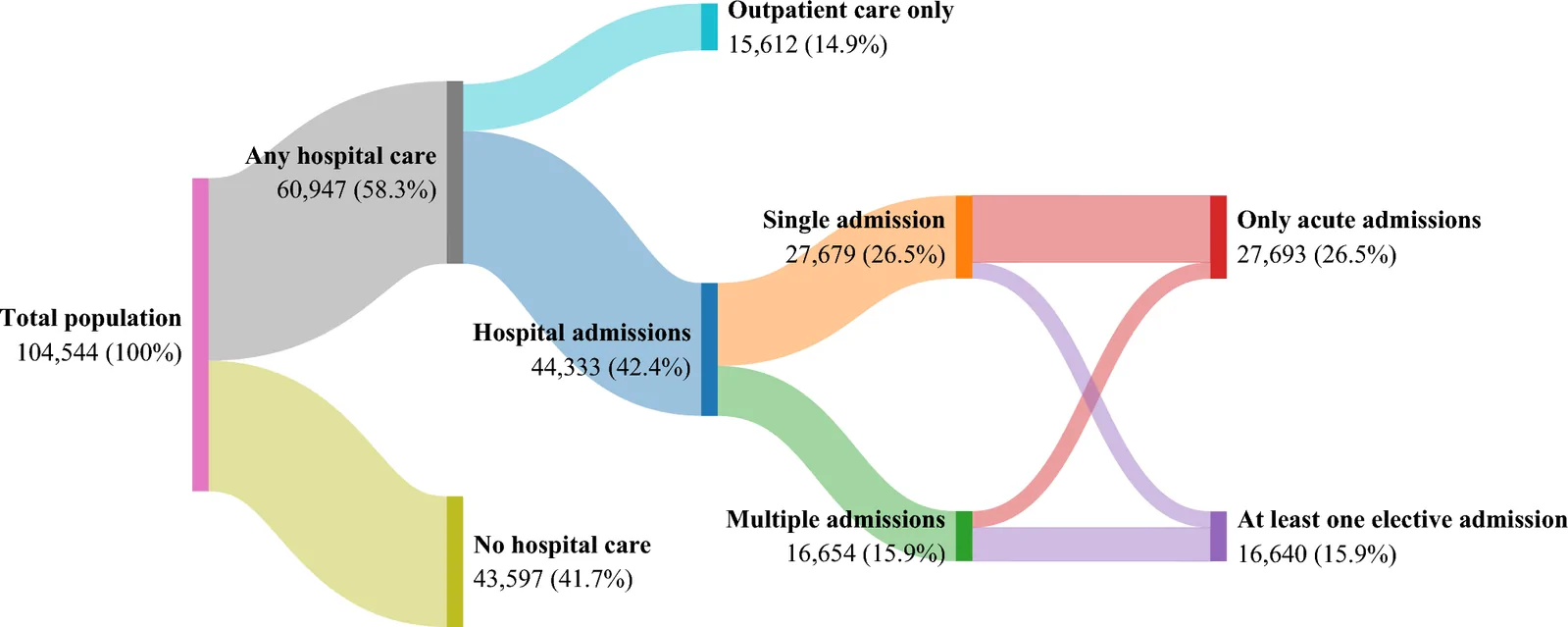
Sankey diagram of hospital care utilization in the final three months of life. Numbers represent individuals and percentages were taken from the total population. Sankey diagram shows proportion of older adults that received hospital care, were admitted to the hospital and were electively admitted to the hospital in the final three months of life

Figure 2 presents a Sankey diagram illustrating in- and outpatient hospital care utilization among older adults three months before their death and identifies those electively admitted in this period. In total 104,544 older adults died between April and December 2019 (median age 83 years [IQR: 75–88], 53,961 (51.6%) female; Table 1). Of the total cohort, 58.3% received hospital care, 14.9% received outpatient care only, and 42.4% were admitted to the hospital. Furthermore, in 15.9% of all individuals at least one elective hospital admission occurred. The older adults that were hospitalized accounted for a cumulative total of 75,425 hospital admissions, of which 28,981 (38.4%) admissions had an elective nature. The elective admissions consisted mainly of one-day admissions (54.2%) and clinical admissions (45.3%).

**Table 1:** Baseline characteristics stratified by being electively admitted in the final three months of life

Characteristics	Total population	Not electively admitted in the final three months of life	Electively admitted in the final three months of life	*p*-values
*N*	104,544	87,904	16,640	
Age in years—median [IQR]	83.00 [75.00, 88.00]	84.00 [77.00, 89.00]	76.00 [71.00, 82.00]	< 0.001
Female sex^a^—no. (%)	53,961 (51.6%)	46,908 (53.4%)	7053 (42.4%)	< 0.001
*Social-economic factors*				
Migration background^b^				< 0.001
No	85,628 (81.9%)	71,155 (80.9%)	14,473 (87.0%)	
1st generation	13,748 (13.2%)	12,493 (14.2%)	1255 (7.5%)	
2nd generation	5168 (4.9%)	4256 (4.8%)	912 (5.5%)	
Income^c^—no. (%)				< 0.001
Low income	26,858 (25.7%)	21,466 (24.4%)	5392 (32.4%)	
Medium income	16,994 (16.3%)	13,059 (14.9%)	3935 (23.6%)	
High income	19,562 (18.7%)	14,402 (16.4%)	5160 (31.0%)	
Unknown/institutionalized	30,959 (29.6%)	28,828 (32.8%)	2131 (12.8%)	
Missing	10,171 (9.7%)	10,149 (11.5%)	22 (0.1%)	
Living alone—no. (%)				< 0.001
No	62,236 (59.5%)	51,383 (58.5%)	10,853 (65.2%)	
Yes	32,135 (30.7%)	26,371 (30.0%)	5764 (34.6%)	
Missing	10,173 (9.7%)	10,150 (11.5%)	23 (0.1%)	
*Psychological/cognitive factors*				
Dementia—no. (%)	25,975 (24.8%)	25,054 (28.5%)	921 (5.5%)	< 0.001
Psychotropic medication—no (%)				< 0.001
No	42,610 (40.8%)	35,653 (40.6%)	6957 (41.8%)	
Yes	35,829 (34.3%)	26,886 (30.5%)	8943 (53.7%)	
Institutionalized	26,105 (25.0%)	25,365 (28.9%)	740 (4.4%)	
*Physical health factors*				
Polypharmacy^d^—no. (%)				< 0.001
No	17,758 (17.0%)	16,952 (19.3%)	806 (4.8%)	
Yes	60,681 (58.0%)	45,587 (51.9%)	15,094 (90.7%)	
Institutionalized	26,105 (25.0%)	25,365 (28.9%)	740 (4.4%)	
Home care and institutionalization^e^—no. (%)				< 0.001
NO	46,617 (44.6%)	35,727 (40.7%)	10,890 (65.4%)	
HH	3688 (3.5%)	2828 (3.2%)	860 (5.2%)	
PC	15,598 (14.9%)	12,813 (14.6%)	2785 (16.7%)	
HH + PC	7743 (7.4%)	6638 (7.6%)	1105 (6.6%)	
NHH	4793 (4.6%)	4533 (5.2%)	260 (1.6%)	
Institutionalized	26,105 (25.0%)	25,365 (28.9%)	740 (4.4%)	
Cause of death—no. (%)				< 0.001
Cancer	26,327 (25.2%)	16,661 (19.0%)	9666 (58.1%)	
Heart disease	16,544 (15.8%)	14,156 (16.1%)	2388 (14.4%)	
Respiratory disease	4329 (4.1%)	3922 (4.5%)	407 (2.4%)	
Renal disease	1065 (1.0%)	870 (1.0%)	195 (1.2%)	
Liver disease	459 (0.4%)	312 (0.4%)	147 (0.9%)	
Alzheimer’s, dementia and senility	12,704 (12.2%)	12,536 (14.3%)	168 (1.0%)	
Neurodegenerative disease	1849 (1.8%)	1,746 (2.0%)	103 (0.6%)	
Cerebrovascular disease	2802 (2.7%)	2621 (3.0%)	181 (1.1%)	
Other	28,352 (27.1%)	24,970 (28.4%)	3382 (20.3%)	
Missing	10,113 (9.7%)	10,110 (11.5%)	3 (0.0%)	
Death in hospital—no. (%)				< 0.001
No	74,330 (71.1%)	63,317 (72.0%)	11,013 (66.2%)	
Yes	20,101 (19.2%)	14,477 (16.5%)	5624 (33.8%)	
Missing	10,113 (9.7%)	10,110 (11.5%)	3 (0.0%)	

^a^Sex as registered in the Personal Records Database (BRP)

^b^1st generation when born abroad, 2nd generation when at least one parent was born abroad

^c^Income relative to the Dutch social minimum, categorized as low (0–139%), medium (140–199%), and high (≥200%)

^d^Polypharmacy was defined as ≥5 drugs dispensed in 2019

^e^NO: no claimed home care; HH: household help; PC: nurse-based personal care; HH+PC: household and nurse-based personal care; NHH: nursing home care at home; Institutionalized: nursing home care in institution

Table 1 shows the baseline characteristics stratified by having at least one elective admission. All baseline characteristics were significantly different between groups. Missing data was imbalanced for several variables, with lower proportions in the electively admitted group. This group was younger (76 vs. 84 years), had less dementia (5.5% vs. 28.5%), and lived independently more frequently (no home care (NO): 65.4% vs. 40.6%; institutionalized: 4.4% vs. 28.9%). Causes of death expected to have needed palliative care were more often recorded in the electively admitted group (79.9% vs. 60.1%), especially cancer (58.1% vs. 19.0%). In-hospital deaths were also more common in this group (33.8% vs. 16.5%).

### Elective treatments

Table 2 shows the 15 selected elective treatments, their frequencies, proportions in the final month of life, and the number of individuals treated. Recurrent treatments were most prevalent, such as chemo- or immunotherapy, radiotherapy, and dialysis. The frequencies of recurrent treatments during elective hospital admissions, and cataract surgery, were underrepresented as many occurred outside of registered elective admissions (Online Resource 3, Table S4). Chemo- and/or immune therapy via infusion was declared 12,102 times in the final three months of life, radiotherapy 13,924 times, dialysis 30,087 times, and cataract surgery 584 times compared to much lower frequencies declared during elective admissions only (Table 2).

**Table 2:** The 15 selected elective treatments occurring during elective admissions in the final three months of life

Treatment		Approach	Number of persons with declared treatment [total = 16,640^a^; *n* (%)]	Total declared treatments [counts (%)]	Declared treatments in final month of life [counts (%)]
*Systemic treatments*					
Chemo- and/or immunotherapy via infusion or injection^b^			2687 (16.1%)	6460	1353 (20.9%)
Radiotherapy^b^			329 (2.0%)	521	165 (31.7%)
*Ophthalmologic surgeries*					
Cataract surgery^b^			149 (0.9%)	176	37 (21.0%)
*Gastro-intestinal (GI) interventions*					
Therapeutic endoscopy GI-tract			787 (4.7%)	979	361 (36.9%)
GI-ostomy surgeries			217 (1.3%)	228	91 (39.9%)
		Open		94 (41.2%)	
		Minimally-invasive		134 (58.8%)	
Resection GI tract organ			196 (1.2%)	229	148 (64.6%)
		Open		155 (67.7%)	
		Minimally-invasive		74 (32.3%)	
Exploratory laparotomy			175 (1.1%)	177	104 (58.8%)
Nephrologic and urologic interventions					
Dialysis			288 (1.7%)	738	299 (40.5%)
		Peritoneal dialysis		80 (10.8%)	
		Haemodialysis		658 (89.2%)	
Cardiovascular interventions					
Heart valve surgery			165 (1.0%)	179	108 (60.3%)
		Open		100 (55.9%)	
		Minimally-invasive		79 (44.1%)	
Coronary artery intervention			237 (1.4%)	266	161 (60.5%)
		CABG^c^		103 (38.7%)	
		PCI^d^		163 (61.3%)	
Aorta and/or direct branches surgery			130 (0.8%)	147	104 (70.7%)
		Open		92 (62.6%)	
		Minimally-invasive		55 (37.4%)	
Peripheral arterial intervention			551 (3.3%)	786	327 (41.6%)
		Open		176 (22.4%)	
		Minimally-invasive		610 (77.6%)	
Amputation/exarticulation leg			104 (0.6%)	115	59 (51.3%)
*Orthopedic surgeries*					
Hip surgeries excl. fractures			116 (0.7%)	136	56 (41.2%)
*Gaining access*					
Gaining access, potentially for feeding			611 (3.7%)	683	327 (47.9%)
		Venous		528 (77.3%)	
		Gastro-intestinal		155 (22.7%)	

^a^Total population of older adults who were electively admitted in the final three months of life

^b^Table only includes treatments declared during elective admissions. For treatments not requiring multiple day admissions, one-day admissions are often not registered. Numbers in this table are therefore an underrepresentation

^c^CABG: coronary artery bypass grafting

^d^PCI: percutaneous coronary intervention

Therapeutic endoscopies of the gastro-intestinal (GI) tract consisted of several different target organs and procedures. Of these, 41.4% represented endoscopic retrograde cholangiopancreatographies (ERCPs). Several persons underwent therapeutic endoscopies at least twice as indicated by the difference between treatment count and individuals (979 treatments in 787 persons). GI tract organ resections included esophagectomies, gastrectomies, small intestine resections, colectomies, and rectum resections. Half of this group (119, 52.0%) was represented by colectomies and the majority of GI tract organ resections used an open approach (67.7%).

Cardiovascular interventions were also prominently featured in the selected elective treatments. Interventions for central cardiovascular diseases included heart valve surgeries, coronary artery interventions (percutaneous coronary interventions (PCIs) and coronary artery bypass grafting (CABGs)), and interventions of the aorta and/or direct branches. These interventions had high proportions occurring in the final month of life, ranging from 60.3% to 70.7%. Among the recorded heart valve surgeries, 55.9% used an open approach. For treating coronary diseases, more PCIs than CABGs (163 vs 103 treatments) occurred.

Peripheral arterial interventions consisted of percutaneous transluminal angioplasties (PTAs), open and endovascular axillo-(bi)femoral, femoral-popliteal or femoral-tibial trajectory interventions, and open and endovascular peripheral artery reconstructions. Endovascular treatments were most common, mainly because of the high occurrence of PTAs (552 treatments).

Care activities possibly representing gaining access for feeding were grouped together. Some care activities specified their use for feeding, others did not. In total, 31 insertions of venous catheters, 56 percutaneous gastro-jejunostomies, and 25 duodenal tubes were intended for artificial nutrition with certainty.

## Discussion

This nationwide study showed 42% of older adults were admitted, and 16% were electively admitted, in the final three months of life. This means almost one in six older adults is at risk of undergoing potentially inappropriate elective treatments in the last phase of their life. Although some treatments may have been intended to improve quality of life, it is unlikely that patients derived meaningful benefit. Patients had a limited life expectancy of only a few months following treatment. In addition, these potentially inappropriate treatments consume time, healthcare resources, and financial costs that could potentially be allocated more effectively elsewhere.

Previous research has consistently shown substantial hospital use in the final months of life, particularly among patients with life-limiting diseases [1–6, 14]. We found that 42% of Dutch older adults were admitted in the final three months of life, whereas disease specific cohorts reported hospitalization rates of 43–63% (likely excluding one-day admissions), and even 9% of being hospitalized twice in the final 30 days of life [3, 14]. To our knowledge, no previous research on end-of-life hospital care has distinguished between urgency or specifically focused on elective care. In our study, 16% of Dutch older adults were electively admitted during the final three months of life. This group often died of a life-limiting disease (e.g., 58% died of cancer), suggesting many deaths were anticipated, and a third of this group died in hospital. This pattern aligns with previous findings that patients with advanced cancer often undergo inappropriate interventions late in life and more often die in hospital [1, 3, 4]. Our findings also show that another 42% of older decedents received no hospital care in the final three months of life, which may be attributable to the careful management and referral practices of general practitioners. Furthermore, only a small proportion of institutionalized persons were electively admitted, suggesting that physicians specializing in elderly care in the Netherlands play a significant role in coordinating and limiting unnecessary hospital admissions. Both general practitioners and elderly care physicians frequently manage complex end-of-life needs outside the hospital setting.

The appropriateness of elective treatments at the end of life should be carefully considered. Western healthcare systems are prone to medical overuse, driven by structural financial incentives, fear of litigation, biases leading to unrealistic hope, and a cultural attitude medicalizing death instead of accepting it as a natural life event [7, 18, 19]. In our cohort, all patients died within three months of treatment, making the time to experience benefits minimal. Many of these treatments have also previously been identified as potentially inappropriate for patients with limited life expectancy. For example, chemotherapy in cancer patients has been labelled aggressive, with timeframes ranging from less than three months to less than 14 days before death [1–3, 5]. Major surgeries, tube feeding, and newly initiated dialysis have also been viewed as inappropriate end-of-life care [1, 6, 15]. Taking a more conservative approach to elective treatments at the end of life could enhance quality of care by creating space for comfort, dignity, and holistic well-being, while also prioritizing quality of life, symptom management, and time with loved ones in accordance with patients’ values and preferences [12, 20, 21]. However, in practice, decisions surrounding invasive or intensive treatments in the older patient are often complex. A key challenge is the timely recognition of the end-of-life phase. Tools to aid in this recognition are being developed (e.g., the Surprise Question), but challenges remain, and physicians tend to overestimate life expectancy [22, 23]. The provision of potentially inappropriate care is driven by this prognostic uncertainty, but also by delayed initiation of palliative care and advance care planning, insufficient shared-decision making, and behavioural factors among physicians, patients, and families [2, 24]. By fostering open conversations about elective treatments at the end of life, clinicians and patients are encouraged to reconsider certain interventions and refrain from them when they are unlikely to provide meaningful benefit.

Our study has several strengths. The use of comprehensive, real-world datasets from Dutch population-level databases enabled us to explore treatment patterns without the constraints of predefined treatments or subgroups, thereby minimizing selection bias. These datasets provided an unique opportunity to investigate the underexplored area of elective care in the final months of life. To ensure transparency, we provided a flowchart and detailed lists of care activities to support our categorization and selection processes. While differences in healthcare practices must be considered, our findings are likely generalizable to older adults in other high-income countries with similar palliative care patterns. The Netherlands is among the countries leading efforts to limit inappropriate end-of-life care, suggesting that elective treatments could be more common elsewhere [3]. A key strength of our study is that it demonstrates how countries with similar nationwide databases can adapt our methodology to identify elective end-of-life care within their own healthcare systems.

Our study also has limitations. We utilized data from 2019, as post-COVID-19 data were not yet available. As healthcare utilization patterns may have changed during and after the pandemic, the findings may not fully reflect current end-of-life hospital care practices. In addition, Dutch population-level databases are primarily designed for administrative and financial purposes and may contain inaccuracies or incomplete data. Because clinical details on the nature of admissions and treatments were unavailable, we relied on proxies. Furthermore, the accuracy of care activity documentation is uncertain, potentially affecting the precision of our results. The exploratory nature of our study required categorization and selection of elective treatments, introducing some subjectivity. Although the efforts were made to ensure consistency, this may have influenced the interpretation of our findings.

## Conclusions

We showed 16% of older adults were electively admitted in the final three months of life, elective hospital care is thus common in this phase. A portion likely conflicts with the goals of care and patients’ wishes for their final months. This study can help start discussions on the appropriateness of elective care at the end of life. Further research should focus on developing strategies to reduce potentially inappropriate elective treatments, such as identifying patient characteristics that can help distinguish patients unlikely to benefit from elective treatments due to limited life expectancy. The overarching goal must be to optimize quality of life at the end of life and ensure care aligns with patients' goals and wishes.

## Supplementary Information

Below is the link to the electronic supplementary material.

Online Resourse 1 (DOCX 50 KB)

Online Resource 2 (DOCX 93 KB)

Online Resource 3 (DOCX 29 KB)
