# Supplementary material for: Elective admissions and treatments in the final three months of life: a nationwide retrospective study of older adults
Source: Eur Geriatr Med. 2026 Aug 3;17(5):2465–74. doi: 10.1007/s41999-026-01560-5 (PMC13754975; doi:10.1007/s41999-026-01560-5)
Supplement: Supplementary file 1 [file 41999_2026_1560_MOESM1_ESM.docx]

**Online Resource 1: Methods resources**

Title:	Elective Admissions and Treatments in the Final Three Months of Life: A Nationwide Retrospective Study of Older Adults

Authors:	Sofie Rutten, Joost W. Van Den Berg, Rob D. Van Der Mei, Bianca M. Buurman, and Hanna C. Willems

Correspondence:	Sofie Rutten, s.rutten2@amsterdamumc.nl, address: Section of Geriatric Medicine, Internal Medicine, Amsterdam University Medical Centre location University of Amsterdam, Meibergdreef 9, 1105 AZ, Amsterdam, the Netherlands

Content:	This file contains supplemental methodological information on utilized registries, study definitions and a flowchart on the classification of health care utilization.

**1.1: List of utilized registries	2**

**1.2: Study definitions, Table S1	4**

**1.3: Health care utilization in the three months preceding death, Figure S1	5**

**References	6**

**1.1: List of utilized registries**

- GBAHUISHOUDENBUS <https://www.cbs.nl/nl-nl/onze-diensten/maatwerk-en-microdata/microdata-zelf-onderzoek-doen/microdatabestanden/gbahuishoudensbus-huishoudenskenmerken> https://www.cbs.nl/nl-nl/onze-diensten/maatwerk-en-microdata/microdata-zelf-onderzoek-doen/microdatabestanden/gbahuishoudensbus-huishoudenskenmerken
- MEDICIJNTAB <https://www.cbs.nl/nl-nl/onze-diensten/maatwerk-en-microdata/microdata-zelf-onderzoek-doen/microdatabestanden/medicijntab-geneesmiddelen-op-atc-code--4-->
- INHATAB <https://www.cbs.nl/nl-nl/onze-diensten/maatwerk-en-microdata/microdata-zelf-onderzoek-doen/microdatabestanden/inhatab-inkomen-van-huishoudens>
- KOPPELPERSOONHUISHOUDEN
- GBAOVERLIJDENTAB <https://www.cbs.nl/nl-nl/onze-diensten/maatwerk-en-microdata/microdata-zelf-onderzoek-doen/microdatabestanden/gbaoverlijdentab-datum-van-overlijden-van-personen-ingeschreven-in-het-gba>
- WLZINTAB <https://www.cbs.nl/nl-nl/onze-diensten/maatwerk-en-microdata/microdata-zelf-onderzoek-doen/microdatabestanden/wlzzintab-personen-metgebruik-wlz-zorg-in-natura>
- GEBWMOTAB <https://www.cbs.nl/nl-nl/onze-diensten/maatwerk-en-microdata/microdata-zelf-onderzoek-doen/microdatabestanden/gebwmotab-personen-met-wmo-maatwerkvoorzieningen>
- ZVWWVPTAB <https://www.cbs.nl/nl-nl/onze-diensten/maatwerk-en-microdata/microdata-zelf-onderzoek-doen/microdatabestanden/zvwwvptab-personen-met-gebruik-van-zvw-wijkverpleging>
- GBAPERSOONKTAB <https://www.cbs.nl/nl-nl/onze-diensten/maatwerk-en-microdata/microdata-zelf-onderzoek-doen/microdatabestanden/gbapersoonktab-persoonskenmerken-beperkt-in-de-brp>
- MSZZorgactiviteitenVEKTTAB <https://www.cbs.nl/nl-nl/onze-diensten/maatwerk-en-microdata/microdata-zelf-onderzoek-doen/microdatabestanden/mszzorgactiviteitenvekttab-zorgactiviteiten-diagnose>
- LBZBASISTAB <https://www.cbs.nl/nl-nl/onze-diensten/maatwerk-en-microdata/microdata-zelf-onderzoek-doen/microdatabestanden/lbzbasistab-ziekenhuisopnamen-lbz>

**1.2: Study definitions**

**Table S1: Study definitions**

| Variable | Definition | Corresponding codes |
| --- | --- | --- |
| Migration background | Dependent on country of birth as stated in Personal Records Database. 1^st^ generation when born in foreign country. 2^nd^ generation when at least one parent was born in foreign country. | NA |
| Sex | Derived from the Personal Records Database (BRP), which is by default the sex assigned at birth. In The Netherlands, individuals aged ≥16 can change their BRP-registered sex when it does not match their gender-identity through the municipality. | NA |
| Income relative to social minimum | Income per household relative to the Dutch social minimum in percentiles. For persons 65 years or older the minimum is equal to the benefit under the General Old Age Act (AOW). Unavailable for unknown year incomes and institutionalized persons. | NA |
| Polypharmacy | When a person was dispensed 5 or more different medications in 2019. | NA |
| Psychotropic medication use | When at least one psychotropic medication was dispensed in 2019. Dispensaries by hospitals during admission and nursing homes are not included. | ATC-4 codes: N05 (psycholeptics) and/or N06 (psychoanaleptics) |
| Home care | Classified as: no claimed home care (NO), household help (HH), personal care (PC), household help and personal care (HH + PC), and nursing home care at home (NHH) ^1^. Persons had to receive at least one day of home care in January 2019. | Household help (HH) – WMO: 006, 007, 100, 101, 102, 103, 104, 105, 107 & 711; Personal care (PC) – ZVW: 1, 2, 3 & 4; Household help + personal care (HH + PC): a combination of an included WMO code & ZVW code; Nursing home care at home (NHH) – WLZ: 2 & 3 |
| Institutionalization | Long-Term Care Act codes corresponding with nursing home care at an institute. Persons had to receive at least one day of nursing home care at an institute in January 2019. | Institutionalized – WLZ: 1, 11 & 12 |
| Causes of death* | By ICD-10 codes from the death registry and categorized into predefined chronic disease groups estimated to have needed palliative care ^2^. | Cancer: C00–C97; Heart disease: I00–I52 (excl. I12 & I13); Respiratory disease: J40–J47, J96; Renal disease: I12, I13, N17, N18, N28; Liver disease: K70–K77; Alzheimer’s, dementia and senility: F01, F03, G30, R54;  Neurodegenerative disease: G10, G12.2, G20, G23.1, G35, G90.3; Cerebrovascular disease: I60–I69 |
| Outpatient care only | Older adults receiving outpatient specialized medical care without being admitted. | CAs: 011005, 011006, 090611, 090612, 090614, 190007, 190010, 190011, 190012, 190013, 190014, 190017, 190019, 190063, 190065, 190067, 190161, 190162, 190163, 411000 |
| Elective hospital admission | We classified an admission as elective when there was no occurrence ED visit or face-to-face contact with an acute nature in other hospital department on the day of or before admission. This was not performed on day admissions as it is not possible to be admitted with this nature from the ED. | CA ED visit (executed by DHD): 190015; CA face-to-face contact with an acute nature in other hospital department (executed by researchers of study): 190016 |

*HIV/AIDS excluded due to small numbers.

**1.3: Health care utilization in the three months preceding death**

Dutch older adults aged ≥65 years who died between 1 April and 31 December 2019

Any care activities declared within 3 months prior to death?

Persons receiving specialized medical care 3 months prior to death

Persons NOT receiving specialized medical care 3 months prior to death

Any out-patient care declarations or hospital admissions?

Persons admitted at least once in the 3 months prior to death

Persons only receiving out-patient care in the 3 months prior to death

How many times was a person admitted in the last 3 months prior to death?

Only one admissions in the 3 months prior to death

Two or more admissions in the 3 months prior to death

Was the nature of at least one admission elective?

At least one elective admission in the 3 months prior to death

Only acute admissions in the 3 months prior to death

**Figure S1 Flowchart determining specialized medical care utilization in the final three months of life**

**References**

1.	Smeekes OS, De Boer, T. R., Van Der Mei, R. D., Buurman, B. M., & Willems, H. C. Receiving home care forms and the risk for emergency department visits in community-dwelling Dutch older adults, a retrospective cohort study using national data. BMC Public Health. 2024;24(1):1792.

2.	S. N. Etkind, A. E. Bone, B. Gomes, et al. How many people will need palliative care in 2040? Past trends, future projections and implications for services. BMC Medicine. 2017;15(102).
