# Supplementary material for: Elective admissions and treatments in the final three months of life: a nationwide retrospective study of older adults
Source: Eur Geriatr Med. 2026 Aug 3;17(5):2465–74. doi: 10.1007/s41999-026-01560-5 (PMC13754975; doi:10.1007/s41999-026-01560-5)
Supplement: Supplementary file 2 [file 41999_2026_1560_MOESM2_ESM.docx]

**Online Resource 2: Care activity selection tables**

Title:	Elective Admissions and Treatments in the Final Three Months of Life: A Nationwide Retrospective Study of Older Adults

Authors:	Sofie Rutten, Joost W. Van Den Berg, Rob D. Van Der Mei, Bianca M. Buurman, and Hanna C. Willems

Correspondence:	Sofie Rutten, s.rutten2@amsterdamumc.nl, address: Section of Geriatric Medicine, Internal Medicine, Amsterdam University Medical Centre location University of Amsterdam, Meibergdreef 9, 1105 AZ, Amsterdam, the Netherlands

Content:	This file contains lists of care activities, their frequencies, and grouping to promote transparency and reproducibility of the selection of elective treatments to highlight.

**Table S2: List A; Care activities with frequency ≥50 and supportive or acute nature	2**

**Table S3: List B; Groups of care activities or independent care activities with frequency ≥50	5**

**Table S2: List A; Care activities with frequency ≥50 and supportive or acute nature**

| **Care activity code** | **Description** | **Frequency** | **Supportive or acute nature** |
| --- | --- | --- | --- |
| 039680 | Perifeer infuus inbrengen. | **8,609** | Yes |
| 039142 | Verstrekking chemotherapie per infuus of per injectie bij gemetastaseerde of hematologische tumoren (excl. acute leukemie zie 039143). | **3,424** | No |
| 039607 | Anesthesie controle door anesthesist op verkoever. | **2,021** | Yes |
| 039605 | Inleiden algemene anesthesie. | **1,823** | Yes |
| 039606 | Uitleiden algemene anesthesie. | **1,780** | Yes |
| 039146 | Verstrekking immunotherapie per infuus of per injectie (excl. desensibilisatie middels immunotherapie bij kinderen zie 039150, excl. behandeling met methotrexaat (MTX) bij kinderen zie 039138). | **1,422** | No |
| 039132 | Procedurele sedatie en analgesie (PSA) buiten de operatiekamer. | **1,134** | Yes |
| 087078 | Abcesdrainage met echografie. | **1,009** | No |
| 039445 | Injecties, met uitzondering van injecties ten behoeve van lokale anesthesie, per zitting. | **903** | No |
| 039145 | Verstrekking chemo-immunotherapie per infuus of per injectie. | **807** | No |
| 039141 | Verstrekking chemotherapie per infuus of per injectie bij niet-gemetastaseerde tumoren. | **541** | No |
| 090791 | Een bestralingsfractie. | **521** | No |
| 039603 | Inbrengen centrale veneuze lijn. | **497** | No |
| 192052 | Hemodialyse met EPO. | **484** | No |
| 039604 | Inbrengen arteriële lijn. | **473** | Yes |
| 090795 | Intensieve image-based positioneringscorrectie. | **442** | Yes |
| 034694 | Endoscopische retrograde cholangiopancreaticografie (ERCP) (zie 034698 voor ERCP met cholangioscopie). | **405** | No |
| 032600 | Behandeling met zuigdrainages van de pleuraholte en het mediastinum. | **402** | No |
| 034394 | Therapeutische endoscopie van oesofagus, maag of duodenum. | **395** | No |
| 033285 | Poliklinische controle op de werking van de pacemaker, inclusief het eventueel gebruik van de cardioverter. | **388** | Yes |
| 039928 | Begeleiding van oncologie patiënten tijdens supportive care/palliatieve zorg zonder chemo- en/of hormoontherapie. | **375** | Yes |
| 080033 | Inbrengen centrale lijn onder echo- of röntgengeleide. | **362** | No |
| 039881 | Urologische behandeling met dilatatie of door katheterisatie, per zitting. | **360** | No |
| 080821 | Percutane transluminale angioplastiek (PTA) stenose van de andere niet-coronaire vaten (zie 080822 voor occlusie). | **305** | No |
| 039608 | Toedienen spinaal anesthesie via katheter - regionale anesthesie. | **299** | Yes |
| 090703 | CT-simulatie. | **283** | Yes |
| 039090 | Algehele anesthesie bij specialistisch onderzoek en bij verrichtingen, waarvoor geen anesthesie staat vermeld. | **267** | Yes |
| 039143 | Verstrekking chemotherapie per infuus of per injectie bij acute leukemie. | **246** | No |
| 039609 | Perifeer block - regionale anesthesie. | **238** | Yes |
| 080830 | Plaatsen stent (vasculair, urinewegen, enteraal, galwegen, traanwegen). | **234** | No |
| 033238 | Acute PTCA ter opheffing/verwijdering stenosen coronaire. | **230** | Yes |
| 038945 | Behandeling wond met wondrandexcisie (o.a. necrotomie, debridement) of wond > 5 cm lengte zonder wondrandexcisie. Incl. onderzoek en schoonmaken en evt. hechten of lijmen. Incl. evt. plaats. verdoven. | **217** | No |
| 039156 | Verstrekking parenteraal ijzer. | **201** | No |
| 039939 | Aerosolbehandeling. | **201** | No |
| 036223 | Transurethrale resectie van een of meerdere grote papillomen en/of maligne blaastumoren. | **198** | No |
| 039163 | Intraveneuze verstrekking van bisfosfonaten. | **193** | No |
| 035512 | Proeflaparotomie. | **177** | No |
| 039600 | Toedienen epiduraal anesthesie via katheter - regionale anesthesie. | **163** | Yes |
| 032688 | Therapeutische pleurapunctie. | **161** | No |
| 035502 | Therapeutische ascitespunctie. | **158** | No |
| 080822 | Percutane transluminale angioplastiek (PTA) occlusie van de andere niet-coronaire vaten (zie 080821 voor stenose). | **158** | No |
| 090706 | 3D planning met intensiteitsgemoduleerde velden (3D-IMRT) (standaard 3D planning zie 090705). | **155** | Yes |
| 039684 | Inbrengen thoraxdrain. | **153** | Yes |
| 031241 | Cataractoperatie extracapsulair, met inbrengen van kunststoflens (zie 031242 voor m.b.v. niet standaard materialen, of uitgevoerd in amblyogene leeftijd). | **140** | No |
| 038944 | Wondbehandeling van een wond kleiner of gelijk aan 5 cm lengte zonder wondrandexcisie met onderzoek en schoonmaken en hechten of lijmen, inclusief eventueel plaatselijk verdoven. | **140** | No |
| 033290 | Behandeling met de cardioverter. | **139** | No |
| 192051 | Hemodialyse. | **136** | No |
| 033266 | Het inbrengen van een stimulatie-elektrode en het aansluiten van een uitwendige pacemaker. | **130** | No |
| 039896 | Begeleiding tijdens de behandeling met immunotherapie, alle toedieningsvormen exclusief per infuus of per injectie (zie 039146) (excl. behandeling met methotrexaat (MTX) bij kinderen zie 039138). | **128** | Yes |
| 034696 | Endoscopisch plaatsen stent in tractus digestivus (proximaal of distaal). | **123** | No |
| 039892 | Begeleiding tijdens behandeling met chemotherapie, alle toedieningsvormen excl per infuus of per injectie (zie 039142), bij gemetastaseerde of hematologische tumoren (excl acute leukemie zie 039893). | **118** | Yes |
| 036170 | Retrograde endoscopische plaatsing van een stent (oa JJ), al dan niet met dilatatie van de ureter. | **103** | No |
| 039951 | Exsanguinatie- of wisseltransfusie, vervolg behandeling. | **102** | No |
| 088198 | Drainwissel na nefrostomie. | **101** | Yes |
| 033670 | Open reconstructie van een perifere slagader door middel van transplantaat, endarteriectomie of patch (zie 033669 voor reconstructie m.b.v. operatiemicroscoop, zie 033668 voor endov.). | **98** | No |
| 090704 | Eenvoudige planning. | **87** | Yes |
| 087658 | Drainageprocedure galblaas of galwegen, met röntgen. | **86** | No |
| 088158 | Nefrostomie bij afvloedbelemmering, met röntgen. | **86** | No |
| 038791 | Amputatie of exarticulatie van een teen. | **83** | No |
| 033289 | Resuscitatie, met intubatie, al of niet met defibrillatie (zie 033288 voor zonder intubatie). | **81** | Yes |
| 039943 | Ambulante behandeling van ulcera cruris door middel van compressietherapie. | **80** | No |
| 038913 | Proefexcisie (stans of mes), al of niet met coagulatie met de hyfrecator exclusief het pathologisch onderzoek. | **79** | No |
| 033105 | Aortocoronaire bypass met 1 arteriële graft, inclusief eventuele veneuze graft(s) en/of kunststof materiaal. | **77** | No |
| 033231 | PTCA eentak ter opheffing/verwijdering stenosen coronaire arterien. | **75** | No |
| 039977 | De eerste acute nierfunctievervangende therapie, bij acuut nierlijden, intoxicaties etc. | **74** | Yes |
| 087258 | Inbrengen maag- of duodenumsonde. | **74** | No |
| 038911 | Operatieve verwijdering van gezwellen uitgaande van cutis, subcutis en/of onderhuids vet- en bindweefsel of verwijderen corpora aliena of inwendige metalen hechtingen e.d. dmv excisie. | **73** | No |
| 039755 | Analyse van een 24-uurs electrocardiografie registratie. | **71** | Yes |
| 033079 | Hartklepvervanging, open procedure. | **68** | No |
| 034503 | Endoscopische gastrostomie (zie 034501 voor open procedure). | **68** | No |
| 039978 | Tweede en volgende nierfunctievervangende therapie, bij acuut nierlijden, intoxicaties etc., in de acute fase. | **68** | Yes |
| 033288 | Resuscitatie, zonder intubatie, al of niet met defibrillatie (excl. met intubatie zie 033289). | **67** | Yes |
| 034738 | Colonresectie, al dan niet met coecostomie, open procedure (zie 034739 voor endoscopisch). | **66** | No |
| 090705 | Standaard 3D planning (3D-IMRT planning zie 090706). | **65** | Yes |
| 038567 | Vervanging van de femurkop en het acetabulum. | **64** | No |
| 033232 | PTCA meertak of hoofdstam ter opheffing/verwijdering stenosen coronaire arterien. | **63** | No |
| 038953 | Vacuüm therapie bij wondbehandeling, inclusief initieel aanbrengen wondbedekker, klinisch, per dag. | **63** | No |
| 032671 | ECC-perfusie per OK sessie, uitgevoerd of standby (inclusief materiaal, inzet hartlong machine en perfusiepersoneel). | **62** | Yes |
| 038590 | Amputatie bovenbeen. | **59** | No |
| 030540 | Neurolytische blokkade van een of meer perifere zenuwen. | **58** | No |
| 033247 | Percutane hartklep implantatie. | **58** | No |
| 033605 | Inbrengen van een port-a-cath systeem. | **58** | No |
| 036210 | Operatief aanleggen van een blaasfistel (suprapubische catheter). | **57** | No |
| 087278 | Percutane gastro- of jejunostomie, de verrichting omvat de punctie, katheterisatie en inbrengen van de katheter. | **56** | No |
| 087678 | Drainageprocedure galblaas of galwegen, met echografie. | **55** | No |
| 033672 | Percutane angioplastiek niet-coronaire perifere arteriën (zie 033351 voor niet-coronaire centrale arteriën). | **54** | No |
| 039897 | Begeleiding van patiënten tijdens de behandeling met radiotherapie. | **54** | Yes |
| 080032 | Lokale injectie medicatie onder echo- of röntgengeleide. | **54** | No |
| 039137 | Verstrekking biologicals per infuus of per injectie bij niet-oncologische diagnosen (excl. bij kinderen zie 039140). | **53** | No |
| 038421 | Plaatsbepaling aandoening met behulp van neuronavigatie. | **52** | Yes |
| 034739 | Endoscopische colonresectie, al dan niet met coecostomie (zie 034738 voor open procedure). | **51** | No |
| 038739 | Amputatie van het onderbeen. | **51** | No |
| 039923 | Begeleiding van patiënten tijdens de behandeling met hormoontherapie bij gemetastaseerde of hematologische tumoren. | **51** | Yes |
| 038853 | Behandeling van grote diepe abcessen zoals mastitis of pectoraal phlegmone, hand- en voetphlegmone, panaritium (uitsluitend met incisie en drainage), perianaal abces en peritonsillair abces. | **50** | No |

**Table S3: List B; Groups of care activities or independent care activities with frequency ≥50**

| **No.** | **Group description** | **Care activity code** | **Description** | **Frequency** |
| --- | --- | --- | --- | --- |
| 1 | Chemo-, immunotherapy by injection or infusion |  |  | **6,460** |
|  |  | 039141 | Verstrekking chemotherapie per infuus of per injectie bij niet-gemetastaseerde tumoren. |  |
|  |  | 039142 | Verstrekking chemotherapie per infuus of per injectie bij gemetastaseerde of hematologische tumoren (excl. acute leukemie zie 039143). |  |
|  |  | 039143 | Verstrekking chemotherapie per infuus of per injectie bij acute leukemie. |  |
|  |  | 039144 | Verstrekking chemotherapie per infuus of per injectie bij niet-oncologische diagnosen. |  |
|  |  | 039145 | Verstrekking chemo-immunotherapie per infuus of per injectie. |  |
|  |  | 039146 | Verstrekking immunotherapie per infuus of per injectie (excl. desensibilisatie middels immunotherapie bij kinderen zie 039150, excl. behandeling met methotrexaat (MTX) bij kinderen zie 039138). |  |
| 2 | Abscess drainage |  |  | **1,078** |
|  |  | 087048 | Abcesdrainage met CT. |  |
|  |  | 087058 | Abcesdrainage met Röntgen. |  |
|  |  | 087078 | Abcesdrainage met echografie. |  |
| 3 | Therapeutic endoscopy gastro-intestinal system |  |  | **979** |
|  |  | 034303 | Endoscopische operatieve behandeling oesophagusstenose (zie 034301 voor open procedure). |  |
|  |  | 034311 | Endoscopische operatieve behandeling oesophagus-divertikel volgens Zenker (zie 034310 voor open procedure). |  |
|  |  | 034321 | Endoscopische radiofrequente ablatie (RF-ablatie) aandoening oesophagus (voor endoscopische mucosectomie zie 034640). |  |
|  |  | 034640 | Endoscopische mucosectomie. |  |
|  |  | 034394 | Therapeutische endoscopie van oesofagus, maag of duodenum. |  |
|  |  | 034395 | Therapeutische endo-echografie van de pancreas (exclusief ETN en ETD, zie 035418 en 035419). |  |
|  |  | 035418 | Endoscopische transluminale necrosectomie (ETN) pancreas (zie 035419 voor ETD pancreas). |  |
|  |  | 035419 | Endoscopische transluminale drainage (ETD) pancreas (zie 035418 voor ETN pancreas). |  |
|  |  | 034695 | Enteroscopie (b.v. enkel- of dubbelballon) met behulp van een flexibele endoscoop inclusief eventuele biopten en poliepectomie. |  |
|  |  | 034696 | Endoscopisch plaatsen stent in tractus digestivus (proximaal of distaal). |  |
|  |  | 034697 | Interventie-coloscopie (behandeling bloeding, dilatatie) niet zijnde stentplaatsing (zie 034696). |  |
|  |  | 034694 | Endoscopische retrograde cholangiopancreaticografie (ERCP) (zie 034698 voor ERCP met cholangioscopie). |  |
| 4 | Peripheral arterial interventions |  |  | **904** |
|  |  | 033673 | Aanleggen axillo-bifemorale bypass. |  |
|  |  | 033674 | Aanleggen axillo-femorale bypass. |  |
|  |  | 033677 | Aanleggen cross-over arteria subclavia of arteria femoralis. |  |
|  |  | 033678 | Aanleggen femoro-popliteale bypass, open procedure (zie 033679 voor endovasculair). |  |
|  |  | 033680 | Aanleggen femoro-tibiale bypass, open procedure (zie 033681 voor endovasculair). |  |
|  |  | 033679 | Endovasculaire behandeling femoro-popliteale traject (zie 033678 voor open procedure). |  |
|  |  | 033681 | Endovasculaire behandeling femoro-tibiale traject (zie 033680 voor open procedure). |  |
|  |  | 033684 | Reconstructie van perifere (slag)ader zonder transplantaat, open procedure (zie 033685 voor endovasculair). |  |
|  |  | 033685 | Reconstructie van perifere (slag)ader zonder transplantaat, endovasculair (zie 033684 voor open procedure). |  |
|  |  | 033668 | Endov.reconstr.van perifere slagader d.m.v.transplant., endarteriectomie, endostent of patch (zie 033669 voor open reconstr.m.b.v. operatiemicr., zie 033670 voor open reconstr.zonder operatiemicr.). |  |
|  |  | 033670 | Open reconstructie van een perifere slagader door middel van transplantaat, endarteriectomie of patch (zie 033669 voor reconstructie m.b.v. operatiemicroscoop, zie 033668 voor endov.). |  |
|  |  | 033682 | Operatie voor perifere aneurysma(ta) of perifere arterioveneuze fistel(s), open procedure (zie 033683 voor endovasculair). |  |
|  |  | 033630 | Operaties aan extra-thoracaal gelegen grote arteriën, zonder herstel der continu‹teit arteriectomie aan de extremiteiten. |  |
|  |  | 033351 | Percutane transluminale angioplastiek niet-coronaire centrale arteriën exclusief de nierarterie (zie 033360). |  |
|  |  | 033672 | Percutane angioplastiek niet-coronaire perifere arteriën (zie 033351 voor niet-coronaire centrale arteriën). |  |
|  |  | 033669 | Open reconstructie van een perifere slagader d.m.v. transplantaat, endarteriectomie of patch m.b.v. operatiemicroscoop (zie 033670 voor reconstructie zonder operatiemicrosc., zie 033668 voor endov.). |  |
|  |  | 080821 | Percutane transluminale angioplastiek (PTA) stenose van de andere niet-coronaire vaten (zie 080822 voor occlusie). |  |
|  |  | 080822 | Percutane transluminale angioplastiek (PTA) occlusie van de andere niet-coronaire vaten (zie 080821 voor stenose). |  |
| 5 | Injections, excluding injections for local anesthesia | 039445 | Injecties, met uitzondering van injecties ten behoeve van locale anesthesie, per zitting. | **903** |
| 6 | Dialysis |  |  | **738** |
|  |  | 192049 | Nachtelijke hemodialyse met epo. |  |
|  |  | 192051 | Hemodialyse. |  |
|  |  | 192052 | Hemodialyse met EPO. |  |
|  |  | 192054 | Thuishemodialyse met EPO. |  |
|  |  | 192056 | Thuishemodialyse met EPO en VDA. |  |
|  |  | 192061 | CAPD inclusief dialysemiddelen, excl. EPO. |  |
|  |  | 192062 | CAPD inclusief dialysemiddelen en EPO. |  |
|  |  | 192063 | CCPD inclusief dialysemiddelen, excl EPO. |  |
|  |  | 192064 | CCPD inclusief dialysemiddelen en EPO. |  |
|  |  | 192068 | Actieve centrum hemodialyse met EPO. |  |
|  |  | 192069 | Passieve centrum hemodialyse met EPO. |  |
| 7 | Gaining access for feeding |  |  | **683** |
|  |  | 039682 | Inbrengen centrale veneuze (voedings)katheter niet gerelateerd aan een operatie. |  |
|  |  | 033698 | Inbrengen centrale veneuze katheter of een navelkatheter bij pasgeborenen voor voeding of drukmeting. |  |
|  |  | 039348 | Plaatsen duodenum voedingssonde met behulp van elektromagnetische transmitter. |  |
|  |  | 087278 | Percutane gastro- of jejunostomie, de verrichting omvat de punctie, katheterisatie en inbrengen van de katheter. |  |
|  |  | 039603 | Inbrengen centrale veneuze lijn. |  |
|  |  | 087258 | Inbrengen maag- of duodenumsonde. |  |
| 8 | Radiotherapy | 090791 | Een bestralingsfractie. | **521** |
| 9 | Treatment with suction drainage of pleural cavity and mediastinum | 032600 | Behandeling met zuigdrainages van de pleuraholte en het mediastinum. | **402** |
| 10 | Urological treatment with dilatation or by catheterization, per session | 039881 | Urologische behandeling met dilatatie of door katheterisatie, per zitting. | **360** |
| 11 | Wound treatment |  |  | **357** |
|  |  | 038944 | Wondbehandeling van een wond kleiner of gelijk aan 5 cm lengte zonder wondrandexcisie met onderzoek en schoonmaken en hechten of lijmen, inclusief eventueel plaatselijk verdoven. |  |
|  |  | 038945 | Behandeling wond met wondrandexcisie (o.a. necrotomie, debridement) of wond > 5 cm lengte zonder wondrandexcisie. Incl. onderzoek en schoonmaken en evt. hechten of lijmen. Incl. evt. plaats. verdoven. |  |
| 12 | Coronary artery intervention |  |  | **266** |
|  |  | 033104 | Aortocoronaire bypass operatie met uitsluitend veneuze graft(s) en/of kunststof materiaal. |  |
|  |  | 033105 | Aortocoronaire bypass met 1 arteriële graft, inclusief eventuele veneuze graft(s) en/of kunststof materiaal. |  |
|  |  | 033106 | Aortocoronaire bypass operatie met 2 arteriële grafts, inclusief eventuele veneuze graft(s) en/of kunststof materiaal. |  |
|  |  | 033107 | Aortocoronaire bypass operatie met 3 of meer arteriële grafts, inclusief eventuele veneuze graft(s) en/of kunststof materiaal. |  |
|  |  | 033231 | PTCA eentak ter opheffing/verwijdering stenosen coronaire arterien. |  |
|  |  | 033232 | PTCA meertak of hoofdstam ter opheffing/verwijdering stenosen coronaire arterien. |  |
|  |  | 033233 | PTCA ter opheffing/verwijdering chronische occlusie coronaire arterien. |  |
|  |  | 033234 | PTCA met passage coronaire arterien graft. |  |
| 13 | Placement stent (vascular, urological, enteral, bile ducts or tear ducts) | 080830 | Plaatsen stent (vasculair, urinewegen, enteraal, galwegen, traanwegen). | **234** |
| 14 | Resection gastro-intestinal tract organs |  |  | **229** |
|  |  | 034323 | Oesophagusresectie, open procedure (zie 034324 voor thoracoscopisch). |  |
|  |  | 034324 | Thoracoscopische oesophagusresectie (zie 034323 voor open procedure). |  |
|  |  | 034399 | Oesophagusresectie met gastric pull-up (met jejunuminterpositie zie 034398, met colonimplantatie zie 034342). |  |
|  |  | 034432 | Maagresectie. |  |
|  |  | 034440 | Totale maagresectie, thoracaal of abdominaal. |  |
|  |  | 034638 | Dunne darmresectie, open procedure (zie 034639 voor endoscopisch). |  |
|  |  | 034639 | Endoscopische dunne darmresectie (zie 034638 voor open procedure). |  |
|  |  | 034733 | Totale colectomie met ileorectale anastomose, open procedure (zie 034735 voor endoscopisch). |  |
|  |  | 034738 | Colonresectie, al dan niet met coecostomie, open procedure (zie 034739 voor endoscopisch). |  |
|  |  | 034739 | Endoscopische colonresectie, al dan niet met coecostomie (zie 034738 voor open procedure). |  |
|  |  | 035024 | Anterior resectie van het rectosigmoid, al dan niet met coecostomie of tijdelijke anus praeternaturalis, open procedure (zie 035025 voor endoscopisch). |  |
|  |  | 035025 | Endoscopische anterior resectie van het rectosigmoid, al dan niet met coecostomie of tijdelijke anus praeternaturalis (zie 035024 voor open procedure). |  |
|  |  | 035026 | Rectum-extirpatie, inclusief aanleggen anus praeternaturalis, open procedure (zie 035027 voor laparoscopisch). |  |
|  |  | 035027 | Laparoscopische rectum-extirpatie, inclusief aanleggen anus praeternaturalis (zie 035026 voor open procedure). |  |
| 15 | Gastro-intestinal ostomy surgeries |  |  | **228** |
|  |  | 034792 | Enterostomie als onderdeel van een laparotomie of om andere redenen, open procedure (zie 034793 voor endoscopisch). |  |
|  |  | 034793 | Endoscopische enterostomie als onderdeel van een laparoscopie of om andere redenen (zie 034792 voor open procedure). |  |
|  |  | 034798 | Enterostomie, open procedure (zie 034799 voor endoscopisch). |  |
|  |  | 034799 | Endoscopische enterostomie (zie 034798 voor open procedure). |  |
|  |  | 034853 | Opheffen van een entero- of ileostomie, open procedure (zie 034854 voor laparoscopisch). |  |
|  |  | 034501 | Gastrostomie, open procedure (zie 034503 voor endoscopisch). |  |
|  |  | 034503 | Endoscopische gastrostomie (zie 034501 voor open procedure). |  |
|  |  | 034796 | Ileostomie, open procedure (zie 034797 voor endoscopisch). |  |
|  |  | 034797 | Endoscopische ileostomie (zie 034796 voor open procedure). |  |
|  |  | 034752 | Aanleggen van een anus preaternaturalis na laparotomie bijvoorbeeld bij ileus of peritonitis, open procedure (zie 034753 voor endoscopisch). |  |
|  |  | 034753 | Endoscopisch aanleggen van een anus preaternaturalis na laparotomie bijvoorbeeld bij ileus of peritonitis (zie 034752 voor open procedure). |  |
|  |  | 034833 | Reconstructie anus praeternaturalis van het colon, intraperitoneaal. |  |
|  |  | 034834 | Reconstructie anus praeternaturalis van het colon, extraperitoneaal. |  |
|  |  | 034850 | Opheffen van anus praeternaturalis door middel van colonresectie, waarna buikwandplastiek. |  |
|  |  | 034851 | Sluiten van een anus praeternaturalis, extraperitoneaal. |  |
| 16 | Aerosol treatment | 039939 | Aerosolbehandeling. | **201** |
| 17 | Iron infusion | 039156 | Verstrekking parenteraal ijzer. | **201** |
| 18 | Transurethral resection bladder tumours/papillomas | 036223 | Transurethrale resectie van een of meerdere grote papillomen en/of maligne blaastumoren. | **198** |
| 19 | Biphosphonate infusion | 039163 | Intraveneuze verstrekking van bisfosfonaten. | **193** |
| 20 | Heart valve surgery |  |  | **179** |
|  |  | 033078 | Hartklepplastiek, open procedure. |  |
|  |  | 033250 | Percutane hartklepplastiek, exclusief katheterdilatatie van hartklep (zie 033241). |  |
|  |  | 033079 | Hartklepvervanging, open procedure. |  |
|  |  | 033247 | Percutane hartklep implantatie. |  |
|  |  | 033248 | Transkatheter hartklepimplantatie, open procedure. |  |
|  |  | 033085 | Thoracoscopische plastiek of vervanging van hartklep. |  |
| 21 | Exploratory laparotomy | 035512 | Proeflaparotomie. | **177** |
| 22 | Cataract surgery |  |  | **176** |
|  |  | 031241 | Cataractoperatie extracapsulair, met inbrengen van kunststoflens (zie 031242 voor m.b.v. niet standaard materialen, of uitgevoerd in amblyogene leeftijd). |  |
|  |  | 031242 | Cataractoperatie extracapsulair, met inbrengen van kunststoflens, m.b.v. niet standaard materialen of technieken, of uitgevoerd in de amblyogene leeftijd. |  |
|  |  | 031251 | Cataractoperatie intracapsulair, met inbrengen van kunststoflens. |  |
|  |  | 031268 | Cataractoperatie extracapsulair. |  |
| 23 | Therapeutic pleural tap | 032688 | Therapeutische pleurapunctie. | **161** |
| 24 | Therapeutic paracentesis | 035502 | Therapeutische ascitespunctie. | **158** |
| 25 | Placement thoraxdrain | 039684 | Inbrengen thoraxdrain. | **153** |
| 26 | Aorta and/or direct branches surgery |  |  | **147** |
|  |  | 033559 | Inbrengen van een aorta-bifurcatie prothese en reconstructie van een arteria renales, open procedure (zie 033560 voor endovasculair). |  |
|  |  | 033560 | Inbrengen van een aorta-bifurcatie prothese en reconstructie van een arteria renales, endovasculair (zie 033559 voor open procedure). |  |
|  |  | 033561 | Inbrengen van een aorta-bifurcatie prothese en reconstructie van beide arteriae renales, open procedure (zie 033562 voor endovasculair). |  |
|  |  | 033562 | Inbrengen van een aorta-bifurcatie prothese en reconstructie van beide arteriae renales, endovasculair (zie 033561 voor open procedure). |  |
|  |  | 033341 | Reconstructie van de aorta of haar directe zijtakken, zoals de arteria subclavia, open procedure (zie 033342 voor endovasculair). |  |
|  |  | 033342 | Reconstructie van de aorta of haar directe zijtakken, zoals de arteria subclavia, endovasculair (zie 033341 voor open procedure). |  |
|  |  | 033554 | Reconstructie aan de aorta of haar directe zijtakken zoals arteria renales en arteria iliaca, open procedure (zie 033555 voor endovasculair). |  |
|  |  | 033555 | Reconstructie aan de aorta of haar directe zijtakken zoals arteria renales en arteria iliaca, endovasculair (zie 033554 voor open procedure). |  |
|  |  | 033089 | TAAA (thoraco-abdominaal aorta aneurysma), open procedure. |  |
|  |  | 033090 | Endovasculaire TAAA (thoraco-abdominaal aorta aneurysma). |  |
|  |  | 033092 | Endovasculaire vervanging aorta descendens thoracalis. |  |
|  |  | 033093 | Vervanging aorta ascendens met aortaboog. |  |
|  |  | 033094 | Vervanging aortaboog. |  |
|  |  | 033095 | Vervanging aortawortel, aorta ascendens en aortaboog. |  |
|  |  | 033096 | Vervanging aortawortel. |  |
|  |  | 033097 | Vervanging aortaklep, aortawortel en aorta ascendens. |  |
|  |  | 033098 | Vervanging aorta ascendens zonder circulatiestilstand. |  |
|  |  | 033099 | Vervanging aorta ascendens met circulatiestilstand. |  |
|  |  | 033155 | Endovasculaire correcties aan de intrathoracale grote arteriën, o.a. aortaruptuur of aneurysma arteria anonyma (zie 033154 voor open procedure). |  |
| 27 | Drainage gallbladder/bile ducts |  |  | **146** |
|  |  | 087648 | Drainageprocedure galblaas of galwegen, met CT. |  |
|  |  | 087658 | Drainageprocedure galblaas of galwegen, met röntgen. |  |
|  |  | 087678 | Drainageprocedure galblaas of galwegen, met echografie. |  |
|  |  | 035340 | Galblaas drainage, open procedure (zie 035342 voor endoscopisch). |  |
|  |  | 035342 | Endoscopische galblaas drainage (zie 035340 voor open procedure). |  |
| 28 | Pacemaker (electrodes) surgeries |  |  | **144** |
|  |  | 033259 | Inbrengen implanteerbare intracardiale pacemaker. |  |
|  |  | 038840 | Subcutaan plaatsen pulsgenerator (neuromodulator, pacemaker) excl. t.b.v. DBS en epidurale stimulatie (zie 030117, 030197 en 039432). (I.h.k.v. SNM-studie zie 032703). |  |
|  |  | 033283 | Vervanging van een pacemaker. |  |
|  |  | 033284 | Verwijdering van een pacemaker. |  |
|  |  | 033267 | Het inbrengen van een stimulatie-elektrode en het aansluiten van een subcutaan geplaatste pacemaker. |  |
|  |  | 033272 | Het inbrengen van twee endocardiale elektroden en het aansluiten van een subcutaan geplaatste pacemaker. |  |
|  |  | 033279 | Het inbrengen van twee endocardiale elektroden en één sinus coronarius elektrode, en het aansluiten en het afregelen van een biventriculaire pacemaker. |  |
|  |  | 033286 | Het bevestigen van een stimulatie-elektrode op het epicard na het openen van het hartzakje en het aansluiten van een subcutaan geplaatste pacemaker. |  |
|  |  | 033254 | Conventionele transveneuze explantatie van endocardiale elektroden van AICD of pacemaker d.m.v. simpele tractie technieken (voor complexe transv. leadextractie AICD of PM zie resp. 033258 of 033257). |  |
|  |  | 033257 | Complexe transveneuze verwijdering van endocardiale elektroden van een pacemaker met gebruik van specifieke extractietools. |  |
| 29 | Amputation foot/toe |  |  | **141** |
|  |  | 038795 | Wegnemen 5e teen plus metatarsale V met uitzondering van de basis. |  |
|  |  | 038791 | Amputatie of exarticulatie van een teen. |  |
|  |  | 038793 | Exarticulatie van de voet. |  |
|  |  | 038794 | Exarticulatie volgens chopart of lisfranc, respectievelijk amputatie transmetatarsaal. |  |
|  |  | 038790 | Amputatie in de voetwortel. |  |
| 30 | Cardioverter treatment | 033290 | Behandeling met de cardioverter. | **139** |
| 31 | Gaining access for long term therapy |  |  | **139** |
|  |  | 033605 | Inbrengen van een port-a-cath systeem. |  |
|  |  | 080832 | Inbrengen implanteerbaar intravasculair systeem (b.v. port-a-cath of dialysekatheter). |  |
|  |  | 039685 | Inbrengen therapiekatheter. |  |
| 32 | Surgical removal dermatological tumor or corpora aliena |  |  | **138** |
|  |  | 038907 | Operatieve verwijdering van gezwel door middel van Moh's chirurgie. |  |
|  |  | 038909 | Operatie van grote en gecompliceerde gezwellen, excl. Moh's chirurgie. |  |
|  |  | 038911 | Operatieve verwijdering van gezwellen uitgaande van cutis, subcutis en/of onderhuids vet- en bindweefsel of verwijderen corpora aliena of inwendige metalen hechtingen e.d. dmv excisie. |  |
|  |  | 038912 | Operatieve verwijdering van gezwellen, corpora aliena en dergelijke, uitgaande van of zich bevindende in dieper liggende structuren dan in code 038911 is omschreven. |  |
| 33 | Hip surgeries excl. fractures |  |  | **136** |
|  |  | 038570 | Vervangen onderdeel van heupprothese. |  |
|  |  | 038565 | Vervanging van de femurkop. |  |
|  |  | 038567 | Vervanging van de femurkop en het acetabulum. |  |
|  |  | 038569 | Verwijderen van een ge‹mplanteerde total hip + re‹mplantatie nieuwe total hip. |  |
|  |  | 038540 | Artrotomie van heupgewricht. |  |
|  |  | 038541 | Capsulectomie en artrolyse van het heupgewricht. |  |
|  |  | 038591 | Exarticulatie van het heupgewricht. |  |
|  |  | 038505 | Verwijdering heuppen. |  |
|  |  | 038568 | Verwijderen total hip bij een niet aan de operatie aansluitende infectie. |  |
|  |  | 038524 | Pseudarthrose operatie fractuur van het collum van het femur door middel van transplantatie of osteotomie. |  |
| 34 | Placement temporary electrodes external pacemaker | 033266 | Het inbrengen van een stimulatie-elektrode en het aansluiten van een uitwendige pacemaker. | **130** |
| 35 | Nephrostomy |  |  | **121** |
|  |  | 036087 | Percutane nefrostomie. |  |
|  |  | 088148 | Nefrostomie bij afvloedbelemmering, met CT. |  |
|  |  | 088158 | Nefrostomie bij afvloedbelemmering, met röntgen. |  |
|  |  | 088178 | Nefrostomie bij afvloedbelemmering, met echografie. |  |
| 36 | Neurolytic block |  |  | **118** |
|  |  | 030540 | Neurolytische blokkade van een of meer perifere zenuwen. |  |
|  |  | 030581 | Neurolytisch splanchnicusblok, onder beeldvormende techniek. |  |
|  |  | 030580 | Neurolytisch thoracaal symphaticusblok, onder beeldvormende techniek. |  |
|  |  | 030583 | Neurolytisch lumbaal sympathicusblok, onder beeldvormende techniek. |  |
| 37 | Amputation leg |  |  | **115** |
|  |  | 038590 | Amputatie bovenbeen. |  |
|  |  | 038591 | Exarticulatie van het heupgewricht. |  |
|  |  | 038690 | Amputatie van het onderbeen. |  |
|  |  | 038691 | Exarticulatie van het onderbeen. |  |
| 38 | Wound treatment with vacuum therapy |  |  | **111** |
|  |  | 038952 | Verwisselen wondbedekker t.b.v. vacuüm therapie. |  |
|  |  | 038953 | Vacuüm therapie bij wondbehandeling, inclusief initieel aanbrengen wondbedekker, klinisch, per dag. |  |
|  |  | 038954 | Vacuüm therapie bij wondbehandeling, inclusief initieel aanbrengen wondbedekker, poliklinisch, per dag. |  |
| 39 | Regional lymph node extirpation |  |  | **104** |
|  |  | 033741 | Regionale lymfklierextirpatie, open procedure (zie 033742 voor endoscopisch). |  |
|  |  | 033742 | Endoscopische regionale lymfklierextirpatie (zie 033741 voor open procedure). |  |
|  |  | 033722 | Stageringslymfadenectomie van het kleine bekken, open procedure (zie 033724 voor laparoscopisch niet retroperitoneaal, zie 033725 voor laparoscopisch retroperitoneaal). |  |
|  |  | 033724 | Laparoscopische stageringslymfadenectomie van het kleine bekken (zie 033722 voor open procedure, zie 033725 voor laparoscopisch retroperitoneaal). |  |
|  |  | 033725 | Laparoscopische retroperitoneale stageringslymfadenectomie van het kleine bekken (zie 033722 voor open procedure, zie 033724 voor laparoscopisch niet retroperitoneaal). |  |
|  |  | 033730 | Therapeutische verwijdering van alle lymfklieren tussen onderkaak en clavicula incl. verwijdering van de musculus sternocleidomastoideus en vena jugularis. |  |
|  |  | 033721 | Uitgebreide verwijdering van para-aortale lymfklieren, open procedure (zie 033723 voor laparoscopisch). |  |
|  |  | 033723 | Laparoscopische uitgebreide verwijdering van para-aortale lymfklieren (zie 033721 voor open procedure). |  |
| 40 | Exsanguination/exchange transfusion |  |  | **104** |
|  |  | 039950 | Exsanguinatie-transfusie, wissel transfusie eerste behandeling. |  |
|  |  | 039951 | Exsanguinatie- of wisseltransfusie, vervolg behandeling. |  |
| 41 | Retrograde placement uro-stent | 036170 | Retrograde endoscopische plaatsing van een stent (oa JJ), al dan niet met dilatatie van de ureter. | **103** |
| 42 | Resection gastro-intestinal organs upper right quadrant |  |  | **102** |
|  |  | 035350 | Cholecystectomie. |  |
|  |  | 035351 | Cholecystectomie inclusief choledochotomie, open procedure (zie 035356 voor endoscopisch). |  |
|  |  | 035355 | Cholecystectomie per laparoscoop, inclusief eventueel peroperatief te verrichten cholangiogram. |  |
|  |  | 035221 | Kwabresectie lever, open procedure (zie 035222 voor laparoscopisch). |  |
|  |  | 035222 | Laparoscopische kwabresectie lever (zie 035221 voor open procedure). |  |
|  |  | 035430 | Pancreatico-duodenectomie volgens Whipple. |  |
| 43 | Treatment ulcera cruris with compressiontherapy | 039943 | Ambulante behandeling van ulcera cruris door middel van compressietherapie. | **80** |
| 44 | Trial excision tissue | 038913 | Proefexcisie (stans of mes), al of niet met coagulatie met de hyfrecator exclusief het pathologisch onderzoek. | **79** |
| 45 | Dialysis shunt surgery |  |  | **69** |
|  |  | 033686 | Aanleggen intraperitoneale shunt ten behoeve van continue ambulante peritoneale dialyse (CAPD), open procedure (zie 033687 voor laparoscopisch). |  |
|  |  | 033687 | Laparoscopisch aanleggen intraperitoneale shunt ten behoeve van continue ambulante peritoneale dialyse (CAPD, zie 033686 voor open procedure). |  |
|  |  | 033691 | Aanleggen uitwendige arterioveneuze shunt. |  |
|  |  | 033692 | Aanleggen inwendige arterioveneuze shunt. |  |
|  |  | 033693 | Aanleggen inwendige arterioveneuze shunt met bypass. |  |
|  |  | 033695 | Opheffen uitwendige arterioveneuze shunt. |  |
|  |  | 033696 | Opheffen inwendige arterioveneuze shunt. |  |
|  |  | 033697 | Reconstructie arteriële of veneuze pool bij in- of uitwendige arterioveneuze shunt exclusief revisie inwendige arterioveneuze shunt (zie 033700 en 033701). |  |
|  |  | 033701 | Revisie inwendige arterioveneuze shunt, open procedure (zie 033700 voor endovasculair). |  |
| 46 | Surgery braintumour/cavernoma |  |  | **62** |
|  |  | 030114 | Operatieve behandeling intraparenchymale aandoening supratentorieel oppervlakkig (oa tumor, cavernoom) zonder betrokkenheid ventrikel, basale kernen, thalamus. |  |
|  |  | 030176 | Operatieve behandeling intraparenchymale aandoening supratentorieel diep (oa tumor, cavernoom) met betrokkenheid 1e,2e of 3e ventrikel, insula thalamus. |  |
|  |  | 030177 | Operatieve behandeling intraparenchymale aandoening infratentorieel diep (oa tumor, cavernoom), bodem 4e ventrikel, medulla oblongata en hersenstam. |  |
|  |  | 030181 | Operatieve behandeling schedelbasis tumor centraal, achterste schedelgroeve, clivus, open procedure. |  |
|  |  | 030184 | Operatieve behandeling intraparenchymale aandoening infratentorieel oppervlakkig (oa tumor, cavernoom ) in cerebellaire hemisfeer. |  |
|  |  | 030189 | Operatieve behandeling tumor schedelbasis frontaal, sphenoid, hypofyse, orbita, transcraniële benadering. |  |
| 47 | Surgical placement supra-pubic bladder fistel/catheter | 036210 | Operatief aanleggen van een blaasfistel (suprapubische katheter). | **57** |
| 48 | Therapeutic bronchoscopy |  |  | **55** |
|  |  | 032484 | Therapeutische bronchoscopie, zoals verwijderen van corpora alinea, afzuigen van secretie of installatie van medicamenten. |  |
|  |  | 032485 | Therapeutische bronchoscopie met laser-coagulatie, zoals bij behandeling van een tumor of bloeding, en / of stentplaatsing. |  |
| 49 | Cystectomy |  |  | **54** |
|  |  | 036240 | Partiële cystectomie, open procedure (zie 036241 voor laparoscopisch). |  |
|  |  | 036241 | Laparoscopische partiële cystectomie (zie 036240 voor open procedure). |  |
|  |  | 036250 | Totale cystectomie, open procedure (zie 036256 voor laparoscopisch). |  |
|  |  | 036252 | Totale cystectomie met urinedeviatie door middel van het implanteren van een of beide ureteren in een geisoleerd darmsegment, in een zitting. |  |
|  |  | 036256 | Laparoscopische totale cystectomie (zie 036250 voor open procedure). |  |
|  |  | 036258 | Totale blaasvervanging met behulp van een geisoleerd darmsegment zonder stoma, inclusief de cystectomie, laparoscopisch (zie 036257 voor open procedure). |  |
| 50 | Removal lung section |  |  | **54** |
|  |  | 032522 | Bilobectomie, open procedure (zie 032523 voor thoracoscopisch, zie 032524 voor VATS procedure). |  |
|  |  | 032530 | Lobectomie of segmentresectie, open procedure (zie 032536 voor thoracoscopisch, zie 032537 voor VATS procedure). |  |
|  |  | 032536 | Thoracoscopische lobectomie of segmentresectie (zie 032530 voor open procedure, zie 032537 voor VATS procedure). |  |
|  |  | 032537 | Lobectomie of segmentresectie met behulp van video-assisted thoracic surgery (VATS, zie 032536 voor thoracoscopisch, zie 032530 voor open procedure). |  |
|  |  | 032542 | Pneumonectomie, open procedure (zie 032547 voor thoracoscopisch, zie 032549 voor VATS procedure). |  |
|  |  | 032543 | Pneumonectomie met uitgebreide verwijdering lymfklieren, open procedure (zie 032548 voor endoscopisch). |  |
|  |  | 032532 | Wigresectie, open procedure (zie 032538 voor thoracoscopisch, zie 032539 voor VATS procedure). |  |
|  |  | 032539 | Wigresectie met behulp van video-assisted thoracic surgery (VATS, zie 032538 voor thoracoscopisch, zie 032532 voor open procedure). |  |
|  |  | 032433 | Sleeve-resectie, open procedure (zie 032434 voor thoracoscopisch, 032435 voor VATS procedure). |  |
| 51 | Hernia inguinalis surgery (open, endoscopic, recurrence) |  |  | **54** |
|  |  | 035700 | Hernia inguinalis, open procedure (zie 035710 voor laparoscopisch). |  |
|  |  | 035710 | Endoscopische operatie hernia inguinalis (zie 035700 voor open procedure. |  |
|  |  | 035703 | Recidief hernia inguinalis, open procedure (zie 035713 voor endoscopisch). |  |
|  |  | 035713 | Endoscopische operatie recidief hernia inguinalis (zie 035703 voor open procedure). |  |
| 52 | Local injection with medication using ultrasound or X-ray | 080032 | Lokale injectie medicatie onder echo- of röntgengeleide | **54** |
| 53 | Biologicals infusion | 039137 | Verstrekking biologicals per infuus of per injectie bij niet-oncologische diagnosen (excl. bij kinderen zie 039140). | **53** |
| 54 | Treatment large deep abscess | 038853 | Behandeling van grote diepe abcessen zoals mastitis of pectoraal phlegmone, hand- en voetphlegmone, panaritium (uitsluitend met incisie en drainage), perianaal abces en peritonsillair abces. | **50** |
