# Supplementary material for: Elective admissions and treatments in the final three months of life: a nationwide retrospective study of older adults
Source: Eur Geriatr Med. 2026 Aug 3;17(5):2465–74. doi: 10.1007/s41999-026-01560-5 (PMC13754975; doi:10.1007/s41999-026-01560-5)
Supplement: Supplementary file 3 [file 41999_2026_1560_MOESM3_ESM.docx]

**Online Resource 3: Treatment occurrence three months prior to death**

Title:	Elective Admissions and Treatments in the Final Three Months of Life: A Nationwide Retrospective Study of Older Adults

Authors:	Sofie Rutten, Joost W. Van Den Berg, Rob D. Van Der Mei, Bianca M. Buurman, and Hanna C. Willems

Correspondence:	Sofie Rutten, s.rutten2@amsterdamumc.nl, address: Section of Geriatric Medicine, Internal Medicine, Amsterdam University Medical Centre location University of Amsterdam, Meibergdreef 9, 1105 AZ, Amsterdam, the Netherlands

Content:	This file contains data on elective treatments and their occurrence during elective admissions, and also the occurrence disregarding admissions.

**Table S4: Comparing treatment occurrence disregarding admissions and occurrence during elective admissions in the final three months of life**

|  | During final three months of life disregarding admissions | | | During elective admissions in the final three months of life | | |
| --- | --- | --- | --- | --- | --- | --- |
| Treatments | Total declared treatments (count) | Declared treatments in final month of life (counts (%)) | Number of persons with declared treatment (n) | Total declared treatments (counts) | Declared treatments in final month of life (counts (%)) | Number of persons with declared treatment (n) |
| Chemo- and/or immunotherapy via infusion/injection | 12,102 | 2,834 (23.4%) | 3,811 | 6,460 | 1,353 (20.9%) | 2,687 |
| Radiotherapy | 13,924 | 3,972 (28.5%) | 2,925 | 521 | 165 (31.7%) | 329 |
| Dialysis | 30,087 | 9,646 (32.1%) | 808 | 738 | 299 (40.5%) | 288 |
| Cataract surgery | 584 | 118 (20.2%) | 475 | 176 | 37 (21.0%) | 149 |
